# Clinical Outcomes and Safety Associated with Adjunctive Antibiotic-Loaded Calcium Sulphate Beads in Hip and Knee Periprosthetic Joint Infection: A Systematic Review

**DOI:** 10.3390/jcm15197509

**Published:** 2026-09-27

**Authors:** Abdulelah K. Alqawlaq, Ruwaydah Hamoud M. Alruways, Wesal Abdulrahman Alqurashi, Ola Mustafa Altaib, Ayman Awlia, Ahmed Bannan, Moayd A. Awad, Bandar Hetaimish, Mohammed R. Algethami, Ramy Samargandi

**Affiliations:** 1 General Medicine Practice Program, Batterjee Medical College, Jeddah 21442, Saudi Arabia; 2 Faculty of Medicine, King Abdulaziz University, Jeddah 21589, Saudi Arabia; 3 Department of Orthopedic Surgery, College of Medicine, University of Jeddah, Jeddah 23218, Saudi Arabia; 4 Department of Medicine, College of Medicine, University of Jeddah, Jeddah 23218, Saudi Arabia; 5 Department of Family and Community Medicine, College of Medicine, University of Jeddah, Jeddah 23218, Saudi Arabia

**Keywords:** periprosthetic joint infection, calcium sulphate beads, local antibiotic delivery, total hip arthroplasty, total knee arthroplasty, revision arthroplasty, DAIR, systematic review

## Abstract

**Background/Objectives:** Antibiotic-loaded calcium sulphate beads (CSBs) are biodegradable local antibiotic carriers used as an adjunct to surgical and systemic antimicrobial treatment of periprosthetic joint infection (PJI). Their incremental clinical benefit, however, remains uncertain. This systematic review aimed to evaluate the effectiveness and safety of adjunctive antibiotic-loaded CSB in hip and knee PJI, with particular attention to outcomes according to surgical strategy. **Methods:** Five electronic databases (PubMed, Scopus, Web of Science, EBSCO, and Google Scholar) were searched from inception to 15 May 2026. Comparative non-randomized studies were assessed using ROBINS-I and non-comparative studies using the Joanna Briggs Institute critical appraisal tool. Comparative outcomes were synthesized according to surgical strategy. Formal meta-analysis was not performed because of clinical and methodological heterogeneity; descriptive aggregated proportions were used only for selected safety outcomes. **Results:** Thirteen studies comprising 859 CSB-treated PJI patients were included, comprising six comparative non-randomized and seven non-comparative observational studies. Infection-control rates in revision-based cohorts ranged from 75.0% to 95.7%, whereas implant-retention cohorts reported more variable success, ranging from 52.0% to 88.0%. No directly comparative one-stage study was identified. Descriptive aggregated proportions for explicitly reported safety outcomes were 4.7% for wound drainage, 5.7% for hypercalcaemia, and 2.1% for heterotopic ossification. Comparative studies had moderate-to-serious risk of bias, while JBI appraisal identified variable methodological limitations among non-comparative studies. **Conclusions:** Available evidence is predominantly observational and does not establish an independent treatment effect attributable to CSB. Comparative DAIR studies have not demonstrated a consistent reduction in reinfection, while comparative evidence for revision strategies remains limited. The incremental clinical benefit of adjunctive CSB remains uncertain, and higher-quality comparative studies with standardized treatment protocols, outcome definitions, and longer follow-up are needed.

## 1. Introduction

Periprosthetic joint infection (PJI) remains one of the most serious complications following total hip arthroplasty (THA) and total knee arthroplasty (TKA), often resulting in repeated surgical interventions, prolonged antimicrobial therapy, functional deterioration, reduced quality of life, and substantial healthcare costs. Although the incidence following primary arthroplasty is relatively low, the increasing volume of joint replacement procedures has led to a growing absolute burden of infection. PJI is now recognized as a leading indication for early revision and a major cause of failure following joint replacement [1,2,3].

Current surgical management of PJI should be individualized according to infection chronicity, implant stability, soft-tissue condition, microbial virulence, and host-related factors [4,5,6,7,8]. In selected acute infections with a stable implant and adequate soft-tissue envelope, implant-retention strategies may be appropriate, most commonly debridement, antibiotics, and implant retention (DAIR). Related implant-retention approaches, including debridement, modular component exchange, component retention, and antibiotics (DECRA) and debridement, antibiotics, bead placement, and implant retention (DABRI), have also been described [9,10,11]. In chronic, recurrent, or refractory infection, implant exchange is generally required and may be performed as a one-stage or two-stage revision [12]. When reconstruction or infection eradication is not feasible, definitive resection arthroplasty, arthrodesis, suppressive antibiotic therapy, or palliative salvage options such as guided persistent fistula may be considered [12,13,14,15]. Despite advances in diagnostic criteria, surgical techniques, and antimicrobial therapy, treatment failure and reinfection remain important clinical challenges, particularly in chronic infection and medically complex patients.

To improve local infection control, increasing attention has been directed toward adjunctive local antibiotic delivery systems. PMMA cement has historically been widely used; however, it has several limitations, including incomplete and variable antibiotic elution, persistence as a non-biodegradable material, and the need for subsequent removal in some cases. In addition, residual PMMA may serve as a foreign-body surface for bacterial adhesion and biofilm formation [9,16,17,18,19].

Antibiotic-loaded calcium sulphate beads (CSBs) have emerged as a biodegradable alternative with advantages including sustained antibiotic release, complete resorption, and effective dead-space management [20,21]. Experimental studies have demonstrated high local antibiotic concentrations and inhibition of bacterial colonization [9,22], supporting their use in revision arthroplasty and other orthopedic infections [23,24,25].

Despite increasing adoption, uncertainty remains regarding whether adding CSB improves infection-control outcomes beyond those achieved with surgery and systemic antimicrobial therapy. CSB is used as an adjunct rather than as an isolated treatment, and the available clinical literature is predominantly observational, with substantial variation in surgical strategy, infection characteristics, local antibiotic formulation, systemic antimicrobial regimens, outcome definitions, and follow-up [25,26,27,28,29,30]. Consequently, favorable outcomes reported in uncontrolled cohorts cannot establish an independent treatment effect attributable to CSB. This systematic review therefore aimed to evaluate clinical infection-control outcomes and safety associated with adjunctive CSB in hip and knee PJI, with particular attention to comparative evidence according to DAIR, one-stage revision, and two-stage revision; follow-up duration; systemic antimicrobial co-interventions; microbiological findings; economic considerations; and CSB-associated complications.

## 2. Materials and Methods

### 2.1. Study Design and Registration

This systematic review was conducted in accordance with the Preferred Reporting Items for Systematic Reviews and Meta-Analyses (PRISMA) 2020 statement [31]. The review protocol was prospectively registered in the International Prospective Register of Systematic Reviews (PROSPERO) prior to data extraction and analysis (CRD420261323408).

### 2.2. Search Strategy

A comprehensive search of PubMed, Scopus, Web of Science, EBSCO, and Google Scholar was conducted from database inception to 15 May 2026. Search terms related to CSB and PJI were combined using Boolean operators (AND/OR) and Medical Subject Headings (MeSH) terms where applicable. The core search concepts included (“calcium sulphate” OR “calcium sulfate” OR Stimulan OR “antibiotic beads”) AND (“periprosthetic joint infection” OR “prosthetic joint infection” OR “arthroplasty infection” OR “hip replacement” OR “knee replacement” OR “revision arthroplasty” OR “hip arthroplasty” OR “knee arthroplasty” OR arthroplasty OR DAIR). In addition, the reference lists of all eligible studies and relevant review articles were manually screened to identify further potentially eligible studies not retrieved through the electronic search.

### 2.3. Study Selection

The study selection process followed the PRISMA framework. After the removal of duplicates, titles and abstracts were independently screened by two reviewers (M.R.A. and R.S.) according to the predefined eligibility criteria. Potentially eligible reports then underwent full-text assessment by the same reviewers. Disagreements were resolved through discussion and consensus.

### 2.4. Eligibility Criteria

Studies were eligible if they included adult patients with confirmed hip or knee PJI undergoing surgical treatment with adjunctive antibiotic-loaded CSB and reported extractable clinical infection-control and/or safety outcomes. Randomized trials, prospective cohorts, retrospective cohorts, matched comparative studies, and case series were eligible. Case reports, narrative reviews, conference abstracts without extractable data, biomechanical or animal studies, studies involving an inappropriate population or intervention, and studies unrelated to hip or knee arthroplasty infection were excluded.

### 2.5. Data Extraction and Outcomes

Data extraction was performed independently by two reviewers (M.R.A. and R.S.) using a standardized predesigned collection form. Extracted variables included first author, publication year, country, study design, sample size and population, mean age, joint involved, surgical strategy, calcium sulphate manufacturer, bead volume and antibiotic formulation, implantation technique, systemic antimicrobial therapy, microbiological findings, follow-up duration, infection-control outcomes, reoperation or recurrent infection, economic data when reported, and complications. Discrepancies were resolved through discussion and consensus.

Primary outcomes were clinical infection-control outcomes, including treatment success, recurrent infection, reinfection, and infection-free survival, as defined by the individual studies. Secondary outcomes included reoperation, persistent wound drainage, hypercalcaemia, heterotopic ossification, synovitis, and other reported CSB-associated adverse events. Comparative bead and control groups were extracted separately whenever possible. Data extraction was restricted to published full-text articles and available supplementary material. Study authors were not contacted for additional unpublished information, missing data were not imputed, and unavailable variables were recorded as “Not reported.”

### 2.6. Risk of Bias and Methodological Quality Assessment

Risk of bias was assessed independently by two reviewers (M.R.A. and R.S.) using design-appropriate tools. Comparative non-randomized studies were assessed using the Risk Of Bias In Non-randomized Studies of Interventions (ROBINS-I) tool [32], and non-comparative case series were assessed using the Joanna Briggs Institute (JBI) Critical Appraisal Checklist for Case Series [33]. ROBINS-I domain-level and overall judgments were categorized as low, moderate, serious, critical, or no information. JBI item-level responses were recorded as yes, no, or unclear. Disagreements were resolved through discussion and consensus.

### 2.7. Data Synthesis

Clinical infection-control outcomes were synthesized according to surgical strategy (DAIR, one-stage revision, and two-stage revision), with comparative studies considered separately from non-comparative cohorts. The feasibility of quantitative meta-analysis was assessed within each surgical subgroup. A formal meta-analysis was not performed because the comparative studies were few and remained clinically and methodologically heterogeneous in PJI classification, surgical protocols, outcome definitions, follow-up duration, systemic antimicrobial therapy, and other co-interventions. Study-specific comparative effect estimates and 95% confidence intervals were reported where available.

When a comparative study did not report an effect estimate but provided sufficient event counts and denominators, an unadjusted risk ratio (RR) with a 95% confidence interval was calculated from the published data. These review-calculated estimates were used for study-level interpretation and were not combined into a summary treatment effect. For selected safety outcomes, descriptive aggregated proportions were calculated only when both an explicit event count and an evaluable denominator were available. Events were summed across contributing studies and divided by the corresponding outcome-specific evaluable population; studies not reporting the relevant outcome were excluded from that denominator. These values are descriptive summaries and were not generated using fixed-effects, random-effects, or inverse-variance meta-analytic models.

## 3. Results

A total of 1875 records were identified in the initial database search. After the removal of duplicates and screening of titles and abstracts, potentially relevant full-text articles were assessed for eligibility. Thirteen studies fulfilled the eligibility criteria and were included in the systematic review [20,25,26,27,29,34,35,36,37,38,39,40,41] (Figure 1).

### 3.1. Study Characteristics

Among the included studies, 859 patients with PJI received CSB. Several cohorts also included mixed aseptic revision populations; these were interpreted separately where appropriate. The included studies were published between 2013 and 2025. Six were comparative non-randomized studies [27,29,35,36,40,41], while seven were non-comparative observational studies [20,25,26,34,37,38,39]. The studies evaluated CSB across DAIR, one-stage revision, two-stage revision, and mixed surgical-strategy cohorts. Several studies were primarily safety studies or included broader revision populations and were interpreted according to the PJI-specific or safety outcomes that were extractable. Both hip and knee arthroplasty infections were represented; some studies enrolled mixed THA and TKA populations, whereas others focused specifically on infected knee or hip cohorts.

Follow-up duration was heterogeneous across studies, ranging from 6-week safety surveillance to a median of 53 months overall in the longest comparative cohort. McPherson et al. [20] required a minimum follow-up of 3 months; Kallala and Haddad [38], Agarwal et al. [39], and Flierl et al. [26] reported mean follow-up durations of 16, 19, and approximately 12.7 months, respectively. Kallala et al. [25] reported a mean follow-up of 35 months (range, 0–78). In Piovan et al. [41], follow-up was significantly shorter in the CSB group than in the control group ((16.1 months; range, 12–37) vs. (30.2 months, range, 12–49; *p* = 0.001)). Tarity et al. [27] reported recurrent infection at 2 years, whereas Sigmund et al. [29] reported the longest summarized comparative follow-up, with a median of 53 months overall (39 months in the CSB group and 85 months in the control group). Several studies described scheduled surveillance but did not provide a summary follow-up duration [34,35,36,37,40]. The main characteristics of the included studies, including study design, surgical strategy, and follow-up duration, are summarized in Table 1.

### 3.2. Bead Characteristics and Implantation Technique

Most studies used STIMULAN® (Biocomposites Ltd., Keele, Staffordshire, UK) as the calcium sulphate carrier. Vancomycin combined with gentamicin or tobramycin was the most frequently reported formulation, although several studies used alternative or culture-directed regimens, including cefazolin, daptomycin, amphotericin B, amikacin, or caspofungin [25,34,37,39]. Beads were generally implanted deeply around prosthetic components, within the medial and lateral gutters in knees, around the acetabulum or proximal femur in hips, intracapsularly, or around temporary spacers during staged revision procedures. Several authors specifically recommended avoiding superficial or subcutaneous placement [20,25,38,41]. Bead preparation methods, antibiotic formulations, and implantation techniques across the included studies are summarized in Table 2.

### 3.3. Infection-Control Outcomes by Surgical Strategy

Three comparative studies evaluated DAIR with adjunctive CSB versus DAIR without CSB. Piovan et al. [41] reported failure in 2/17 CSB-treated patients and 3/15 controls (review-calculated RR 0.59, 95% CI 0.11–3.06), with study-reported treatment success of 88% versus 80% (*p* = 0.522). Tarity et al. [27] reported recurrent-infection failure at 2 years in 9/20 versus 5/20 patients (review-calculated RR 1.80, 95% CI 0.73–4.43; *p* = 0.21). Sigmund et al. [29] reported reinfection in 37/102 versus 29/74 patients (OR 0.9, 95% CI 0.5–1.6) and no significant difference in infection-free survival (HR 1.1, 95% CI 0.7–1.8). Thus, comparative DAIR studies did not demonstrate a consistent incremental benefit associated with adjunctive CSB.

Comparative evidence for two-stage revision was limited. Sun et al. [36] reported infection control in 45/47 CSB-treated patients versus 38/47 controls (95.7% vs. 80.9%; review-calculated RR for success 1.18, 95% CI 1.02–1.38; study-reported *p* < 0.05). Iorio et al. [40] reported no recurrence in 20/20 patients receiving a modified two-stage protocol versus 19/20 controls (review-calculated RR for success 1.05, 95% CI 0.95–1.16); however, the intervention also incorporated methylene-blue-guided extensive debridement and other protocol modifications, preventing attribution of the observed outcome specifically to CSB. Dimofte et al. [35] reported an overall cure rate of 42/45 with CSB versus 10/31 without CSB, but the cohort mixed DAIR and two-stage procedures with significantly different strategy distributions between groups; procedure-specific treatment effects were therefore not considered reliably estimable. No directly comparative one-stage revision study was identified.

Among non-comparative PJI cohorts with clearly extractable infection-control outcomes, Agarwal et al. [39] reported resolution in 3/4 patients, Kallala and Haddad [38] reported no clinical evidence of infection at final follow-up in 14/15 patients, and Flierl et al. [26] reported success in 17/33 acute PJI episodes treated with DAIR and adjunctive CSB. These uncontrolled outcomes were interpreted descriptively because concomitant surgery and systemic antimicrobial therapy, together with the absence of a comparator, preclude estimation of the incremental effect of CSB. McPherson et al. [20], Lum et al. [34], Kallala et al. [25], and Sandiford et al. [37] were not used to derive a PJI-specific infection-control proportion because their cohorts were mixed, were primarily safety-focused, or lacked a clearly defined durable PJI effectiveness denominator.

Longer-term comparative evidence remained sparse. Tarity et al. [27] found no significant reduction in recurrent infection at 2 years, while Sigmund et al. [29] reported 5-year infection-free survival of 59% with CSB and 58% without CSB, with no significant difference in infection-free survival (HR 1.1, 95% CI 0.7–1.8). Studies with only a few months of follow-up were therefore interpreted as reporting early or short-term infection control rather than durable eradication [26,27,29].

Systemic antimicrobial therapy was an important co-intervention and varied substantially across studies. Kallala and Haddad [38] administered 6 weeks of organism-sensitive parenteral therapy; Flierl et al. [26] used organism-specific intravenous treatment for at least 6 weeks followed by variable oral therapy; Piovan et al. [41] used 2 weeks of parenteral therapy followed by oral antibiotics; and Tarity et al. [27] administered pathogen-specific intravenous therapy for at least 6 weeks. Sigmund et al. [29] used empirical intravenous therapy followed by microbiology-directed treatment for approximately 3–6 months, while Sun et al. [36], Iorio et al. [40], and Dimofte et al. [35] incorporated study-specific systemic regimens into their revision protocols.

Staphylococcal species were frequently represented, but pathogen-stratified comparative evidence was limited. Flierl et al. [26] reported failure in 10/20 (50%) staphylococcal infections and 6/13 (46%) non-staphylococcal infections (*p* = 0.835). Tarity et al. [27] found no significant between-group difference in microbiological distribution (*p* = 0.38) or antibiotic-resistant organisms (*p* = 0.54), and Sigmund et al. [29] reported a broad microbiological spectrum without pathogen-specific CSB effect estimates. Sun et al. [36] reported that both CSB-group failures involved methicillin-resistant Staphylococcus aureus (MRSA); because this observation was based on only two events, it does not establish reduced CSB effectiveness against MRSA.

Economic outcomes were infrequently reported. Piovan et al. [41] reported an acquisition cost of approximately €3000 for 20 cc of calcium sulphate beads. Tarity et al. [27] reported approximate acquisition costs of US$1200 for 10 cc and US$2000 for 20 cc and raised concerns regarding cost-effectiveness in the absence of significant clinical benefit. Kallala and Haddad [38] reported an approximate cost of £450 for 10 cc of calcium sulphate beads but explicitly stated that cost was not formally investigated. Iorio et al. [40] suggested that shorter treatment and reimplantation intervals may reduce healthcare costs; however, no formal health–economic analysis was performed. No included study reported a formal cost-effectiveness or cost-utility analysis. Study-level infection-control and safety outcomes are summarized in Table 3, while comparative infection-control outcomes stratified by surgical strategy are presented in Table 4.

### 3.4. Descriptive Safety Outcomes

Complication reporting was inconsistent across the included studies, and not every study systematically assessed each adverse event. Descriptive aggregated proportions were therefore calculated only from studies that explicitly reported the relevant event and an evaluable denominator. These values characterize reported complications associated with CSB exposure and should not be interpreted as meta-analytic estimates or as PJI-specific complication risks because several large safety cohorts included broader revision populations.

Wound drainage or leakage was reported in 60 of 1269 evaluable CSB-exposed procedures or patients, corresponding to a descriptive aggregated proportion of 4.7%. Hypercalcaemia was reported in 53 of 934 evaluable CSB exposures (5.7%). In the largest prospective safety cohort, Kallala et al. [25] reported wound drainage in 32/755 procedures (4.2%) and hypercalcaemia in 41/755 (5.4%), including two symptomatic cases [20,25,29,34,35,37,38,41].

Heterotopic ossification was reported in 27 of 1260 evaluable CSB exposures, corresponding to a descriptive aggregated proportion of 2.1%. Kallala and Haddad [38] reported hypercalcaemia in 3/15 patients, including one symptomatic patient who had received 40 cc of CSB, while Sigmund et al. [29] reported hypercalcaemia in 9/102 CSB-treated patients, including one symptomatic case. These descriptive proportions should be interpreted cautiously because bead volume, surgical indication, complication definitions, and surveillance duration varied across studies [25,29,36,37,38,41].

### 3.5. Risk of Bias and Methodological Appraisal

Among the six comparative non-randomized studies, the overall ROBINS-I judgment was moderate for Tarity et al. [27], Sigmund et al. [29], and Sun et al. [36], and serious for Piovan et al. [41], Iorio et al. [40], and Dimofte et al. [35]. The principal concerns were non-randomized treatment allocation, residual confounding, differences in follow-up, imbalance in surgical strategy, and additional treatment co-interventions. The seven non-comparative studies were appraised separately using the JBI case-series checklist; item-level concerns included incomplete reporting, uncertain consecutive or complete inclusion in some cohorts, small sample sizes, heterogeneous populations, and a primary safety rather than effectiveness focus in several studies [20,25,26,34,37,38,39]. JBI appraisal findings were not interpreted as directly equivalent to ROBINS-I risk categories. The ROBINS-I assessments of comparative non-randomized studies are presented in Figure 2, while the JBI critical appraisal of non-comparative studies is shown in Figure 3.

## 4. Discussion

The principal finding of this systematic review is that the available evidence does not establish a consistent independent clinical benefit from adding antibiotic-loaded CSB to otherwise comparable treatment for hip or knee PJI. Although favorable infection-control outcomes were reported in several observational cohorts, CSB was consistently used as part of multimodal treatment that included surgical intervention and systemic antimicrobial therapy. Consequently, outcomes from uncontrolled studies cannot be attributed specifically to CSB [26,27,29,36,38,39,40,41].

The biological rationale for local antibiotic delivery remains plausible. Biofilm formation is a major challenge in PJI because organisms embedded within biofilm demonstrate increased tolerance to antimicrobial therapy and host defenses. Experimental studies have shown that antibiotic-loaded calcium sulphate can achieve high local antibiotic concentrations and inhibit bacterial colonization; however, these mechanistic advantages do not necessarily translate into an independent clinical benefit [4,9,19,20].

Differences in outcomes across surgical strategies may partly reflect the degree of surgical source control rather than an isolated effect of CSB. Revision procedures involve component removal and more extensive debridement, which may reduce residual biofilm burden and create a more favorable environment for adjunctive local antibiotic delivery. In contrast, retained prosthetic components during DAIR may continue to harbor biofilm, potentially limiting the incremental benefit of CSB. Nevertheless, because of available comparative studies being non-randomized and subject to treatment-selection bias, differences in surgical protocols, and additional co-interventions, the apparently more favorable outcomes reported in some revision cohorts should not be interpreted as evidence of an independent strategy-specific benefit of CSB [27,29,35,36,40,41].

Systemic antimicrobial therapy represents another important co-intervention when interpreting the available evidence. Antibiotic regimens differed substantially among the included studies with respect to intravenous treatment, transition to oral therapy, total duration, and pathogen-directed selection. Microbiological findings were also insufficiently consistent to support pathogen-specific conclusions regarding CSB effectiveness. These differences contribute to the clinical heterogeneity of the available literature and further limit attribution of treatment outcomes specifically to local antibiotic beads [26,27,29,35,36,40,41].

The main safety concerns associated with CSB were wound drainage, hypercalcaemia, and heterotopic ossification. Although these events were generally uncommon, their reported frequency varied among studies, and the available observational evidence does not establish a precise dose–risk relationship. Differences in bead volume, implantation technique, surgical indication, patient characteristics, and surveillance methods may all influence complication reporting. These findings support careful implantation technique and appropriate postoperative monitoring, particularly for clinically relevant calcium disturbances [25,29,38].

CSBs should also be considered within the broader range of local antibiotic-delivery strategies. Antibiotic-loaded polymethylmethacrylate (PMMA) remains widely used but is non-resorbable and has limitations related to antibiotic compatibility and elution. Other resorbable and bioactive carriers are also under investigation [42]. Direct intra-articular antibiotic infusion represents another local-delivery approach; however, comparative evidence demonstrating an incremental clinical advantage remains limited [43]. Current evidence therefore does not establish the superiority of CSBs over alternative local antibiotic-delivery strategies.

The present systematic review has limitations. No randomized controlled trial was identified; all six comparative studies were non-randomized and had moderate-to-serious ROBINS-I judgments, while seven studies were non-comparative observational cohorts. Additional limitations included small sample sizes, heterogeneous PJI classifications and surgical strategies, differences in systemic and local antimicrobial regimens, variable definitions of treatment success or recurrence, and substantial variation in follow-up duration. Several studies were primarily designed to evaluate safety or included broader septic and aseptic revision populations. These limitations preclude strong causal inference regarding the independent effect of CSB. More detailed patient-level subgroup analyses according to age, comorbidity, host status, joint involved, and infection chronicity were not feasible because these variables were inconsistently reported across the included studies.

Future research should prioritize prospective, adequately powered comparative studies stratified by treatment pathway, including acute postoperative DAIR, acute hematogenous DAIR, one-stage revision, and two-stage revision. Standardized reporting of bead volume, local antibiotic composition, systemic antimicrobial therapy, implantation technique, microbiological characteristics, wound complications, patient-reported outcomes, and sufficiently long follow-up would improve comparability and help determine whether CSB provides an incremental clinical benefit.

## 5. Conclusions

Antibiotic-loaded calcium sulphate beads may have a role as an adjunct in selected cases of hip and knee PJI, particularly when used with revision procedures that provide effective surgical source control. However, their incremental benefit during DAIR remains uncertain. Reported complications were generally uncommon, although wound drainage and hypercalcaemia remain important safety considerations. Careful patient selection, appropriate bead volume, deep placement, and postoperative monitoring may help reduce complications. Higher-quality comparative studies are needed to better define the optimal indications for CSB use.

## Figures and Tables

**Figure 1:**
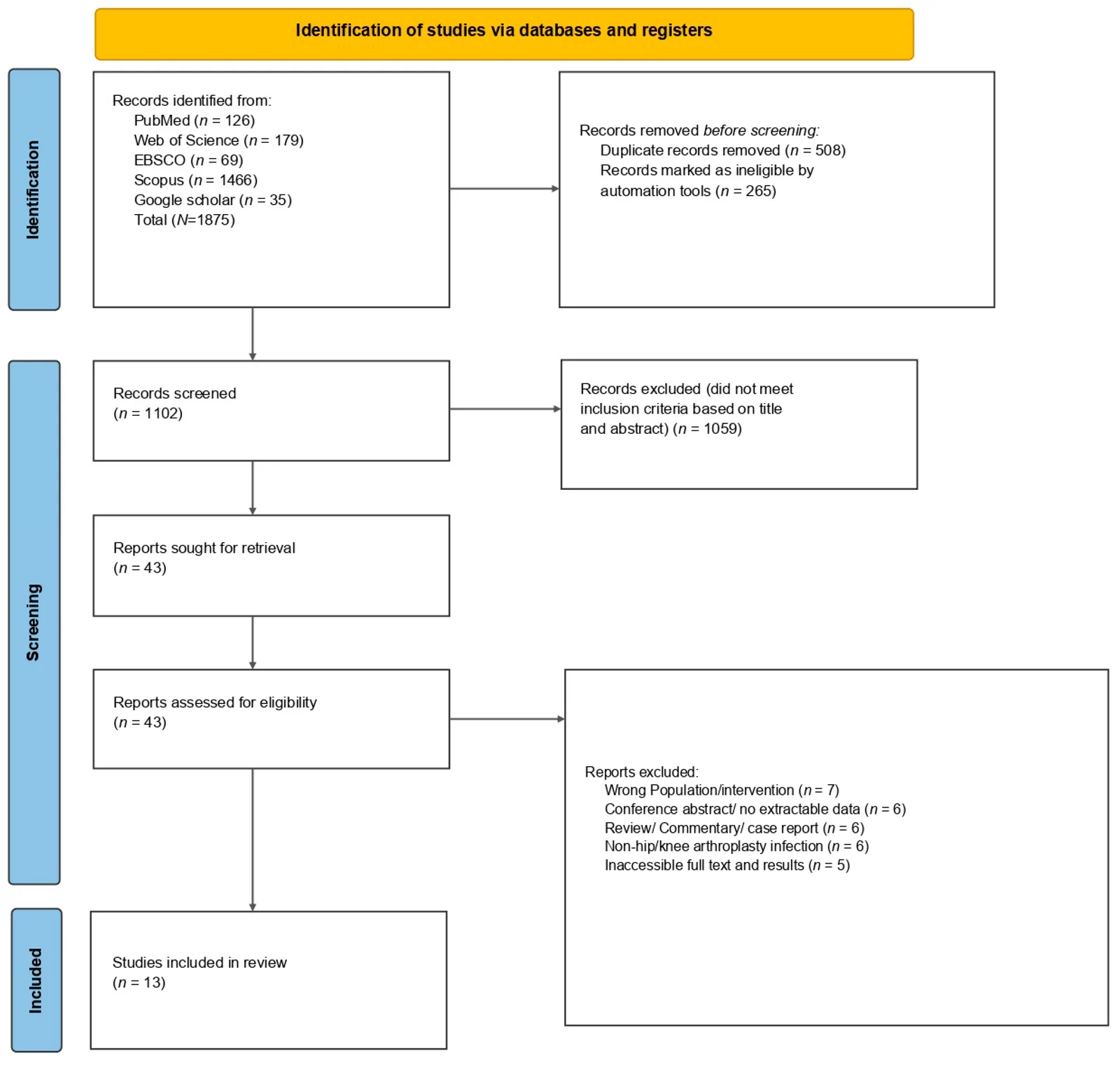
PRISMA 2020 flow diagram of the literature search and study selection process. The diagram summarizes the identification, screening, eligibility assessment, and inclusion of studies evaluating antibiotic-loaded calcium sulphate beads in the management of periprosthetic joint infection (PJI).

**Figure 2:**
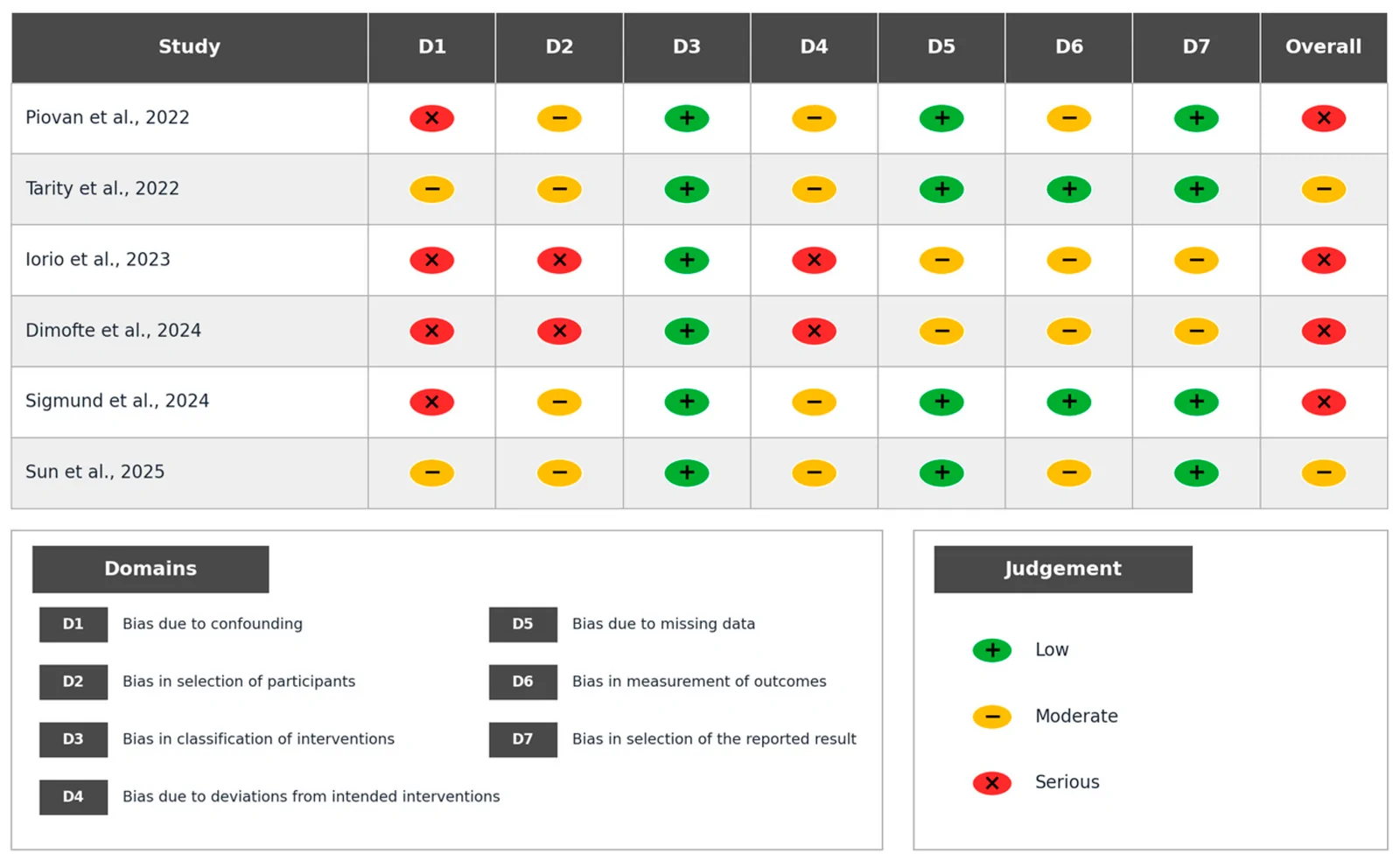
ROBINS-I [32] traffic-light assessment of comparative non-randomized studies: Piovan et al. [41], Tarity et al. [27], Iorio et al. [40], Dimofte et al. [35], Sigmund et al. [29], and Sun et al. [36]. D1, bias due to confounding; D2, bias in selection of participants; D3, bias in classification of interventions; D4, bias due to deviations from intended interventions; D5, bias due to missing data; D6, bias in measurement of outcomes; D7, bias in selection of the reported result. Green indicates low risk, yellow indicates moderate risk, and red indicates serious risk. No study was judged to have a critical risk of bias.

**Figure 3:**
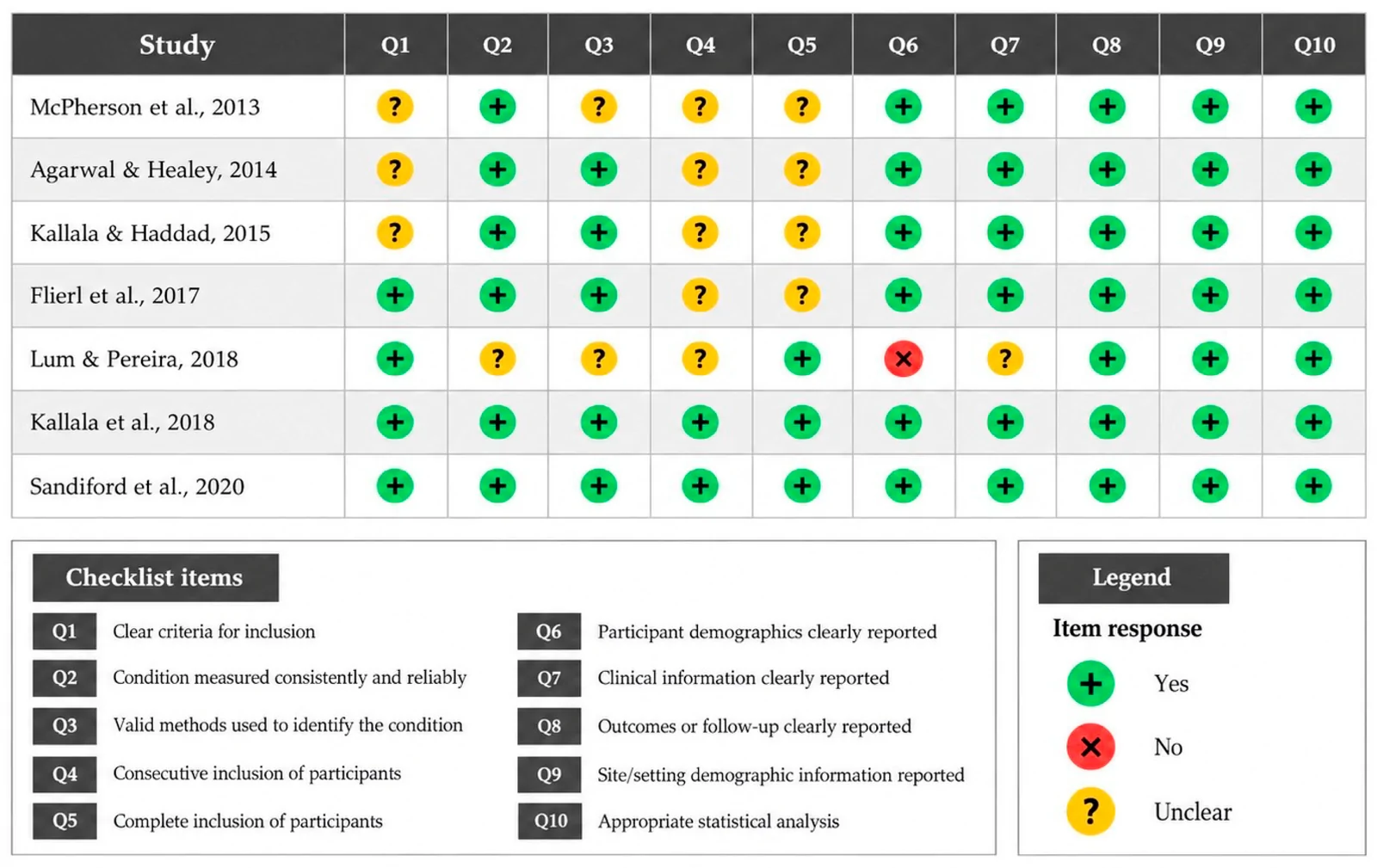
Joanna Briggs Institute (JBI) Critical Appraisal Checklist for Case Series [33] traffic-light assessment of the non-comparative studies: McPherson et al. [20], Agarwal and Healey [39], Kallala and Haddad [38], Flierl et al. [26], Lum and Pereira [34], Kallala et al. [25], and Sandiford et al. [37]. Q1–Q10 represent the JBI checklist items shown in the figure. Green indicates Yes, red indicates No, and yellow indicates Unclear. The JBI item-level appraisal is presented separately from ROBINS-I judgments and is not interpreted as an equivalent risk-of-bias scale.

**Table 1:** Study characteristics of included studies using calcium sulphate beads.

Study	Design/Population	CSB PJI *n*	Surgical Strategy	Follow-Up
McPherson 2013 [20]	Retrospective uncontrolled cohort; *n* = 250 (126 septic/PJI-related)	126	Mixed septic/aseptic revision; DECRA; resection; reimplantation	Minimum 3 months; surveillance at 3, 6, and 12 months
Agarwal 2014 [39]	Retrospective case series; *n* = 4	4	Complex infected hip/knee arthroplasty; revision/removal with reimplantation or fusion	Mean 19 months; range not reported
Kallala & Haddad 2015 [38]	Observational case series; *n* = 15	15	Mixed revision procedures for PJI, including one- and two-stage treatment	Mean 16 months (range 12–22)
Flierl 2017 [26]	Retrospective case series; 32 patients/33 PJI episodes	33	DAIR for acute postoperative or acute hematogenous PJI	Mean 12.7 months (range 3–30); minimum 3 months or until failure
Lum 2018 [34]	Retrospective mixed cohort; *n* = 56; 14 infected revisions	14	High-risk primary/revision arthroplasty	Visits at 2, 6, and 12 weeks and annually; summary duration not reported
Kallala 2018 [25]	Prospective observational safety cohort; *n* = 755; 387 revisions for infection	387	One-stage; DAIR; first- and second-stage revision; mixed indications	Mean 35 months (range 0–78)
Sandiford 2020 [37]	Prospective consecutive hip-PJI series; *n* = 29	29	One-stage, first-/second-stage, DAIR, and other procedures	6-week safety monitoring; no durable infection-control endpoint
Piovan 2022 [41]	Retrospective comparative cohort; *n* = 32	17	DAIR ± CSB for acute post-TKA PJI	CSB 16.1 months (12–37) vs. control 30.2 (12–49); *p* = 0.001
Tarity 2022 [27]	Retrospective matched cohort; *n* = 40	20	DAIR ± CSB for acute/acute hematogenous hip or knee PJI	2-year recurrent-infection outcome
Iorio 2023 [40]	Comparative cohort; *n* = 40	20	Two-stage TKA revision; CSB within modified protocol	1 year after second-stage reimplantation
Dimofte 2024 [35]	Retrospective comparative hip-PJI cohort; *n* = 76	45	DAIR and two-stage exchange	Follow-up scheduled through 12 months and annually; summary duration not reported
Sigmund 2024 [29]	Retrospective comparative cohort; *n* = 176	102	DAIR for early postoperative, acute hematogenous, and chronic PJI	Median 53 months overall; 39 CSB vs. 85 control
Sun 2025 [36]	Retrospective matched cohort; *n* = 94	47	Two-stage revision for chronic primary TKA PJI	Visits at 1, 3, and 6 months and annually; summary duration not reported

CSB, calcium sulphate bead; PJI, periprosthetic joint infection; DAIR, debridement, antibiotics, and implant retention; DECRA, debridement, modular exchange, component retention, antibiotics. “Not reported” indicates that information was unavailable in the published full text or accompanying supplementary material; missing data were not imputed.

**Table 2:** Preparation methods and implantation techniques of calcium sulphate beads across included studies.

Author/Year	Bead Manufacturer	Mixing Procedure and Antibiotics	Implantation Technique
McPherson 2013 [20]	Stimulan^®^	1 g vancomycin per 10 cc calcium sulphate + 240 mg liquid tobramycin; cured in mould.	Intra-articular placement before closure; knees: medial/lateral gutters; hips: deep space inferior to acetabulum and around proximal femur; subcutaneous placement avoided.
Agarwal 2014 [39]	Stimulan^®^	Antibiotic beads prepared intraoperatively, typically vancomycin 1 g per 10 cc powder; occasional daptomycin; molded after mixing.	After final irrigation, larger beads placed in joint space and smaller beads inserted into the medullary canal when applicable.
Kallala & Haddad 2015 [38]	Stimulan^®^	1 g vancomycin + 240 mg gentamicin per 10 cc calcium sulphate with sterile water; set in sterile mould.	Distributed around the joint, prosthesis, or spacer before closure; subcutaneous placement avoided.
Flierl 2017 [26]	Stimulan^®^	10 cc calcium sulphate mixed with 1 g vancomycin + 1.2 g tobramycin.	Implanted during DAIR procedures; exact placement not specified.
Lum 2018 [34]	Stimulan^®^	1 g vancomycin + 1.2 g tobramycin + 1 g cefazolin powder.	Placed within the surgical wound at final closure.
Kallala 2018 [25]	Stimulan^®^	1 g vancomycin per 10 cc calcium sulphate + 240 mg liquid tobramycin; amphotericin B used for fungal infection.	Positioned around prosthetic components or spacers; knees: medial/lateral gutters; hips: deep to acetabulum and around proximal femur.
Sandiford 2020 [37]	Stimulan^®^	Case-specific formulations included vancomycin, gentamicin, amikacin, and caspofungin, mixed according to manufacturer instructions.	4.8-mm beads inserted intracapsularly in hip joint and capsule closed over them; subfascial/subcutaneous placement avoided.
Piovan 2022 [41]	Stimulan^®^	10 cc calcium sulphate mixed with vancomycin 1 g + gentamicin 240 mg or tobramycin 240 mg.	Placed around knee prosthesis, especially medial/lateral gutters and suprapatellar pouch; subcutaneous placement avoided.
Tarity 2022 [27]	Dissolvable antibiotic-loaded calcium sulphate beads	10 cc calcium sulphate contained 1 g vancomycin + 1.2 g tobramycin; larger packs used proportional quantities when reported.	During DAIR, beads placed intra-articularly, commonly in suprapatellar pouch and medial/lateral gutters.
Iorio 2023 [40]	Stimulan^®^	10 mL kit mixed with antibiotics according to antibiogram, e.g., vancomycin 1000 mg + gentamicin 320 mg.	Implanted during first stage of two-stage knee revision after placement of antibiotic spacer.
Dimofte 2024 [35]	Stimulan^®^	Vancomycin 2 g + gentamicin 12 mL mixed in 20 cc Stimulan.	Placed in prosthesis chamber and deep periacetabular regions.
Sigmund 2024 [29]	Stimulan^®^	10 mL rapid-cure calcium sulphate mixed with 1 g vancomycin + 240 mg gentamicin.	After irrigation, beads distributed intra-articularly: hips around inferior acetabulum/proximal femur; knees in medial/lateral gutters and posterior capsule.
Sun 2025 [36]	Stimulan^®^	Culture-directed formulations were used according to organism and susceptibility, including regimens for Gram-positive, Gram-negative, fungal, mycobacterial, or culture-negative infection.	During first stage of two-stage TKA revision, beads placed in suprapatellar pouch and around distal femur and proximal tibia as adjunct to spacer.

THA, total hip arthroplasty; TKA, total knee arthroplasty; DAIR, debridement, antibiotics, and implant retention.

**Table 3:** Study-level infection-control and safety outcomes of calcium sulphate beads.

Study	Strategy	Infection-Control Outcome	Key CSB Complications	Interpretation
McPherson 2013 [20]	Mixed septic/aseptic revision	No clean PJI-specific denominator	Drainage 8/250; HO 3/250; hypercalcaemia not reported	Mixed uncontrolled cohort; no PJI-specific CSB effect estimate
Agarwal 2014 [39]	Complex revision	3/4 (75%) resolution	Endpoint-specific drainage/hypercalcaemia/HO not reported	Very small uncontrolled series
Kallala & Haddad 2015 [38]	Revision for PJI	14/15 (93.3%) without clinical infection	Drainage 0/15; hypercalcaemia 3/15 (1 symptomatic); HO 1/15	Uncontrolled heterogeneous revision cohort
Flierl 2017 [26]	DAIR	17/33 (51.5%) success; 16/33 failure	Endpoint-specific complications not reported	High failure rate; short follow-up may underestimate later failures
Lum 2018 [34]	Mixed primary/revision	No separate PJI effectiveness endpoint	Drainage 1/56; postoperative calcification 1/56	Safety/context only; mixed cohort
Kallala 2018 [25]	Mixed revision; safety cohort	PJI-specific effectiveness not reported	Drainage 32/755; hypercalcaemia 41/755 (2 symptomatic); HO 13/755	Large prospective safety cohort
Sandiford 2020 [37]	Mixed hip-PJI revision	Durable infection-control endpoint not reported	Drainage 1/29; no symptomatic hypercalcaemia; HO 0/29	Early safety study
Piovan 2022 [41]	DAIR ± CSB	15/17 vs. 12/15 success; *p* = 0.522	Drainage 0/17; hypercalcaemia 0/17; HO 0/17	No significant benefit; CSB follow-up shorter
Tarity 2022 [27]	DAIR ± CSB	Failure 9/20 vs. 5/20 at 2 years; *p* = 0.21	Endpoint-specific CSB complications not reported	No clinically meaningful improvement
Iorio 2023 [40]	Two-stage; bundled protocol	20/20 vs. 19/20 without recurrence	No major CSB complications; individual endpoints not reported	CSB effect cannot be isolated
Dimofte 2024 [35]	Mixed DAIR/two-stage	42/45 vs. 10/31 cured; *p* < 0.00001	Wound externalization 1/45; hypercalcaemia 0/45; HO 0/45	Severe confounding and strategy imbalance
Sigmund 2024 [29]	DAIR	Reinfection 37/102 vs. 29/74; OR 0.9 (0.5–1.6)	Drainage 17/102; hypercalcaemia 9/102 (1 symptomatic); HO 8/102	No significant overall benefit
Sun 2025 [36]	Two-stage revision	45/47 vs. 38/47 infection control; *p* < 0.05	HO 2/47; DVT 1/47 vs. 3/47 controls	Favorable retrospective comparison; long-term benefit uncertain

CSB, calcium sulphate bead; PJI, periprosthetic joint infection; DAIR, debridement, antibiotics, and implant retention; DVT, deep-vein thrombosis; HO, heterotopic ossification. “Not reported” indicates that an endpoint-specific value was unavailable and was not imputed.

**Table 4:** Comparative infection-control outcomes stratified by surgical strategy.

Study/Strategy	CSB Group	Control Group	Effect Estimate	Key Limitation
Piovan 2022 [41]—DAIR	2/17 failures	3/15 failures	RR 0.59 (95% CI 0.11–3.06); success *p* = 0.522	Review-calculated RR; shorter CSB follow-up
Tarity 2022 [27]—DAIR	9/20 failures	5/20 failures	RR 1.80 (95% CI 0.73–4.43); *p* = 0.21	Review-calculated RR; no significant benefit
Sigmund 2024 [29]—DAIR	37/102 reinfections	29/74 reinfections	OR 0.9 (0.5–1.6); infection-free survival HR 1.1 (0.7–1.8)	Original-study estimates; historical controls
Iorio 2023 [40]—two-stage	20/20 without recurrence	19/20 without recurrence	RR for success 1.05 (0.95–1.16)	Review-calculated RR; bundled modified protocol
Sun 2025 [36]—two-stage	45/47 infection control	38/47 infection control	RR for success 1.18 (1.02–1.38); *p* < 0.05	Review-calculated RR; retrospective study
Dimofte 2024 [35]—mixed	42/45 cured overall	10/31 cured overall	Procedure-specific effect not reliably estimable	DAIR/two-stage imbalance and treatment-selection confounding

CSB, calcium sulphate bead; DAIR, debridement, antibiotics, and implant retention; RR, risk ratio; OR, odds ratio; HR, hazard ratio; CI, confidence interval. Review-calculated RRs were derived from published event counts and denominators and were not reported by the original investigators. No summary pooled treatment effect was calculated.

## Data Availability

All data analyzed in this review are reported in the article and its tables.
